# The Dental Quarterly

**Published:** 1862-10

**Authors:** 


### THE DENTAL QUARTERLY

Is a neat, spirited young journal, edited by Dr. Ambler Tees, and published by Johnson & Lund, of Philadelphia; the third number of which has just reached us. It originated, we believe, as an advertising medium ; but it is fast becoming something more than this. It is filled with good matter; each number is worth twice the subscription price to any dentist; indeed, about the only fault we find is that it is too much for so little. We hope it may grow and take rank as one of *the* dental journals. We greet its advent with much pleasure. T.

